# Southern Medical Reports: Consisting of General and Special Reports of the Medical Topography, Meteorology and Prevalent Diseases in the Following States: Louisiana, Alabama, Mississippi, North Carolina, South Carolina, Georgia, Florida, Arkansas, Tennessee and Texas; to Be Published Annually

**Published:** 1850-07

**Authors:** 


SOUTHERN MEDICAL REPORTS: *Consisting of General and Special Reports of the Medical Topography, Meteorology and Prevalent Diseases in the following States: Louisiana, Alabama, Mississippi, North Carolina, South Carolina, Georgia, Florida, Arkansas, Tennessee and Texas; to be published annually.* Edited by E. D. FENNER, M. D., of New Orleans, Member of the American Medical Association, &c. &c. Vol. I. 1850. New Orleans, B. M. Norman; New York, Samuel S. and Wm. Wood. pp. 472. 8vo.

Another valuable contribution to a knowledge of the Medical Topography and Diseases of the South and West. The design and general character of these Reports, are highly creditable to their editor, Dr. Fenner. He proposes to fill up in detail the great outlines so ably sketched by Dr. Drake, in the work noticed in our last number; and for this purpose he has appealed to his medical brethren in that region, with a success of which the present volume furnishes a gratifying proof. For vague generalities, speculations, and not seldom declamation too, are now to be substituted detailed descriptions of the required facts, and their legitimate deductions, expressed in plain prose. If figures are introduced, they will be those of arithmetical and algebraical formulæ rather than of poetry.

Doctor Fenner, in his Introductory Address, shows the wide field and the ample materials and necessity, also, for careful and connected histories of the etiology, characteristic phenomena and treatment of the diseases of that vast region, of which he is himself a denizen,—"already inhabited by *millions* of human beings, and capable of maintaining *tens of millions*." While paying a merited compliment to the distinguished physicians of which the South could boast, a century ago, he laments "the long lapse of silent indolence which followed their departure;" and then he asks: "Where shall we look for the records of the deadly pestilence which decimated the inhabited portion of the Southern States half a century ago, or even twenty-five years? With the exception of a few detached essays scattered through the periodicals of our more literary brethren of the North, there is scarcely anything to be found." But, of late, a happy change has come o'er the spirit of their dream.

"Since 1844 we have had as many as four medical journals in the South at one time, three of which are still sustained with much ability, lacking nothing but a better pecuniary support to insure their continuance. Since that period works on practical medicine have, also, recently emanated from Professor S. H. Dickson, of South Carolina, and Doctors Fort and M'Gown, of Georgia."

The contents of this the first volume of Dr. Fenner's proposed annual, in the form of Reports, are Reports from *Louisiana, Alabama, Georgia, Mississippi, Tennessee, South Carolina, and Texas*, together with *Excerpta* and *Miscellanies*, and notices of *Medical Colleges of the South and West*, and of *American Medical Journals.* Some of these papers have appeared before, in the Journals of the region to which the descriptions are confined. Alluding to these periodicals, the editor says: "He expects to select from them whatever they may contain of importance, without lessening their influence or checking their progress. Having been four years connected with one of them, and thus become familiar with their arduous labors, one can but wish them all manner of success."

These Reports manifest the characteristic traits of Southern medical literature, in their being full of talent, written in a flowing and exuberant style, and marked by plausible and ingenious reasoning, but tinctured, withal, by a love of hypothetical disquisition, and straining after remote analogies; facts being too often overlaid by speculative commentaries, and opinions replacing actual history. The writers are not fully aware of the rich materials at their disposal, nor are they conscious of their powers to interest and instruct by a methodical arrangement and exhibition of these materials, to the eschewing of scrappy literature, and attempts to harmonize their own views with those of their distant cotemporaries and famed predecessors.

The reputation of Sydenham awaits him who shall describe the successive epidemic and fixed diseases of New Orleans, with the care and acumen evinced by that great man in his histories of those of London during his own time. The fields of observation turned to such admirable account by Cleghorn in the island of Minorca, and by Hillary in that of Barbadoes, were more limited than those which are within the ken of a physician of Alabama or of Georgia; while the means of insuring more accurate details of meteorology and medical topography can be enjoyed to a much greater extent by an American writer at the present day, than were accessible to the English authors whom we have named.

In offering this little piece of criticism and advice, we ought to add, that the volume before us gives abundant proof, on the part of our medical brethren of the South, not only of an earnest desire to begin a course of careful observation and legitimate inquiry, but, also, of their having made some progress in it. Let us verify this assertion by a more distinct reference to the "Reports."

The first are from Louisiana, and of these the initial article, headed "General Report of the Medical Topography and Meteorology of New Orleans, with an Account of the Prevailing Diseases during the year 1849," is by the Editor. The distant reader will have a good idea of the peculiarly low situation of New Orleans, when he is told :—

"The site being far below the annual elevation of the river, and the *back part* below the occasional rising of the lake, the city has to be protected from inundation by means of *levées* or dams—one running its whole length in front, one above extending from near the river to the swamp, and another covering the entire rear. The levée in front is about seven feet in height—above and in the rear it is not required to be so high. Lake Pontchartrain is situated about five miles back of the city, and its level is about seven feet below the base of the front levée. The intermediate space between the lake and the city is a cypress swamp, presenting, about mid-way, a considerable elevation called the *Metairie Ridge*."

The recession of the river from the city has been of late years considerable, so as to leave a large surface of ground for wharves and stores. The Mississippi rises in March and June.

"The streets of New Orleans run at right angles, and are generally narrow in the older part of the city. Those more recently laid off were allowed much greater width. These are principally above Canal street, and in the rear. In the populated part of the city, at the present time, there are three streets of extraordinary width; viz., Canal, Esplanade, and Rampart. The two former separate the three Municipalities, and the latter intersects them in the rear. These streets are about one hundred feet wide, allowing ample pass way on their sides and handsome promenades in the centres, shaded with trees. There are six public squares, appropriately situated and answering the valuable purposes of recreation and ventilation. We have no recollection of any blind alleys in the city. Situated, as we are, at a convenient distance from the Gulf of Mexico, and under the full influence of its balmy breezes, our city does not suffer in the least from want of proper ventilation. According to Dr. Barton, 'the prevailing winds are, during *winter*, E., N., and N. W.; during *spring*, E., S., and S. E.; during *summer*, S. E., E., and S.; during *autumn*, N., E., and N. E. A perfectly calm atmosphere is very rarely noted; resulting, no doubt, from our alternations of land and water, and the rapid current of the Mississippi before so large a surface of the city.'"

About one half of the inhabited part of the city is paved, and the proportion thus improved is increased every year.

"The streets of New Orleans are proverbially muddy and filthy, owing chiefly to the dampness of the climate and the inefficient means resorted to for cleansing. The dependence for *washing* them, is upon heavy rains, the water-works hydrants, placed at long intervals, and the river when above the level of the city. By means of culverts, the water of the river when high is conducted through the levée into the cross streets, and runs in a rapid stream to the rear of the city, whence it is removed by the Draining Company. It will thus appear that, as yet, we have resorted to nothing but *surface draining;* but Dr. Barton has attempted to show that, notwithstanding the low level of the locality, it is perfectly amenable to effective *underground drainage.*"

On the subject of the supply of water and underground moisture, we are tempted to quote the following:

"The Water Works Company was chartered in 1833, and commenced supplying the city in 1837. It consists of a reservoir three hundred and twenty feet square, erected upon an artificial mound twenty-five feet high, situated in the upper part of the city, two squares from the river. The water is thrown from the river into this reservoir by a powerful steam engine, and thence distributed throughout the city by means of iron tubes placed under ground in the middle of the streets. The extent of these tubes at this time is about thirty-seven miles. From these main tubes are issued branches of leaden tube, which convey the water into houses for all domestic purposes. We were informed at the office of the company, that the extent of leaden tube at this time is about *one million feet.* In our special report on *Colic*, the probable influence of these leaden pipes on the water imbibed in the city is briefly examined. The Water Works Company do not afford a supply of water nearly adequate to the purposes of domestic use and washing the streets. Within a few years past, a plan was submitted to the General Council, by which continued streams were to be kept running at all seasons along the cross streets from the river to the swamp; but it was not adopted. Such a measure might exercise a beneficial influence upon the health of the city. At present, the open gutters often present the most disgusting aspect and are exceedingly offensive to the olfactories. There is still a considerable number of vacant lots in the city, many of which are lower than the level of the streets, and, during wet weather, contain stagnant water, which breeds myriads of musquitoes and evolves deleterious effluvia. Stagnant water is also to be found under many houses."

On the score of prison hygiene we learn, "that the inmates suffer less from the prevailing diseases, such as yellow fever, cholera and the like, than any other persons in the city. The prisons are all situated on the outskirts of the city, which we would suppose were the most unhealthy localities; and the only striking differences between the living of their inmates and the citizens at large, consist in the *regularity and temperance of their habits and their seclusion from the direct rays of the sun.*"

"The privies of New Orleans are necessarily very shallow—extending only about four feet into the ground. According to municipal ordinance, at a late hour of the night their contents are removed and emptied into the river, instead of being deposited in the rear of the city, as formerly.

The greater part of the city is now lighted by *gas*, the works for the preparation of which are situated in the back part of the city, between Gravier and Perdido streets. Their pipes now extend over a distance of more than thirty-four miles."

Differing on this point from Dr. Barton, to whose labors the editor gives frequent credit, the latter says that his own observations have led him to the conclusion that there is a rainy season in New Orleans as regularly as there is frost in winter, or there are swallows in summer. This occurs in the months of June and July. There has been a great increase of rain during the last four years.

*Acclimation.*—"Some do not become *seasoned* until they have suffered the severest form of endemic fever belonging to the climate and locality; others become gradually and thoroughly acclimated without ever suffering an open or severe attack. There are persons who have resided in New Orleans twenty years without ever having had yellow fever, whilst others have had it two or three times. Strong attacks of the severest forms of our remittent, bilious and yellow fever seem to cause a *modification of the system*, which secures to the individual a greater or less immunity from subsequent attacks. Attacks of the *milder* forms, as ordinary intermittents, effect no such immunity; but, on the contrary, when frequent, lead to permanent engorgement of the spleen, and cause an increased liability to the complaint."

Severe attacks of yellow fever, or even mild ones during severe epidemic seasons, certainly give, in Dr. Fenner's opinion, immunity from subsequent attacks, especially if the parties continue to dwell in the same locality. He admits, however, that some persons may be attacked a second time; but, he adds: their system has been so fortified by even a partial acclimation, that they more readily survive the attack than the unacclimated.

Contrary to the common belief, Dr. Fenner shows that *creoles* or *natives* of New Orleans have been attacked with yellow fever. We give his conclusions:

"1. That persons coming from more northern latitudes to this, have to undergo an *acclimation* or *seasoning*, before they become secure in the enjoyment of good health.

2. That this acclimation may be attained without sickness; but that, most generally, it requires the endurance of one or more spells of the customary endemic fevers.

3. That an attack of the endemic yellow fever effects greater security against subsequent attacks, than any form of fever seen in the country; but that the remark is applicable, in some degree, to all of them, excepting the ordinary mild intermittents:

4. That persons may have yellow fever more than once, though it is evident that those who have had one plain attack, usually have little or nothing to dread from subsequent attacks:

5. That *creoles*, or natives of New Orleans, may have yellow fever—though generally, they have it in a very mild form."

So much for our present views of acclimation. The subject is full of interest, and we shall probably recur to it, from year to year, as our experience is enlarged."

After referring to Dr. Barton's annual Report of the Board of Health of New Orleans, and the laborious investigation of the mortality of the city, by Dr. J. C. Simonds, the editor makes the following remarks:

"It will be seen from these reports, that the *sanitary condition* of the city is still *very bad*, and the *mortality very great*. Let us not deceive ourselves, nor 'lay the flattering unction to our hearts,' that we are blest with as fair a portion of health and longevity as falls to the lot of any other city of equal size ; but rather let us look the facts sternly in the face, and endeavor to find a remedy for every existing evil. We shall never commence the great business of reform, until we have become fully aware of our real condition. Little or nothing has ever yet been done *directly* and *expressly* with the view to improving the sanitary condition of the city of New Orleans. True, we have derived, *incidentally*, considerable benefit from improvements established for the purpose of facilitating commerce ; but we trust the time is not far distant, when this intelligent community, enlightened by the investigations of the medical profession, will adopt all necessary measures for rendering our city, as it may be, both a pleasant and safe abode, at all seasons of the year."

Yellow fever depending, as Dr. Fenner believes, on something within the city corporation may, in his opinion, be finally eradicated. Reference is made to the late inundation, of which a fuller account is given in the following part of the article now under notice, also still more in Mr. Farraday's paper subjoined. The editor gives next *a monthly account of the general aspect of the weather, the stage of the river, the condition of the streets, and the principal diseases prevailing in the city of New Orleans, for the year* 1849.

The *naïve* acknowledgment of the editor, touching the fiscal view of professional labor, is worth repeating.

"The year (1849) has been a very unfavorable one to the physicians of New Orleans. During the first part, we had an immense deal of labor and care, with but poor compensation ; and in the middle and later parts, we had but little practice, and of course, small remuneration."

Articles II and III are on the *Inundations* of New Orleans in the years 1816 and 1849, respectively. The latter, by Mr. G. C. Faraday, describes, also, the *Hydrography of the Mississippi River.* The mean depth of the river at Carrolton, nine miles below New Orleans, has been, for thirty years, on an average, 64.4 feet, supposing the bottom to have remained unchanged.

*Annual Report of The New Orleans Board of Health*, constitutes the fourth article of these Reports. As our extracts will necessarily, owing to our small space for inserting them, convey but a very imperfect idea of the scope and variety of topics embraced in the Report, we deem it an act of simple justice to repeat here the introductory remarks of the editor.

"The following report, drawn up by the indefatigable Dr. Barton, one of the committee to whom the task was assigned, will doubtless be read with much interest. We venture to say that no other person in the city would have gone to the trouble of supplying the great amount of valuable statistics, to be found in the tables appended to this report, without the most liberal compensation. Yet here it is—gotten up at *immense labor*, and without any pecuniary remuneration whatever. We cannot expect a continuation of such favors without pay: no man can afford to do it. Dr. Barton seems to have a genius and fondness for those minute observations in meteorology and hygiene, which are so necessary to enable us to arrive at *scientific truth.* They require much time and care, but cannot be dispensed with. We consider the medical profession and the entire community, greatly indebted to Dr. Barton for the beautiful charts and valuable tables accompanying this report, and sincerely regret our inability to insert his large and beautiful chart, on account of the heavy expense."

Dr. Barton begins his report (for the year 1849) by an emphatic declaration of the necessity, under various aspects, of correct and full sanitary returns, which shall exhibit the diseases as modified "by sex, color, length of residence, occupation and particular locality, the actual climate, and the value of life, or expectation of living." "With a reputation abroad for perennial pestilence, with a boasting at home of unparalleled salubrity, it is high time the truth should be known." The ratio of mortality in New Orleans seems to have been at length ascertained.

The mortality from the class of *Fevers* was 14.58 of the entire mortality, of which more than the half (or 55.30 per cent.) is from *yellow fever.*

Of deaths from *pulmonary* diseases among the fixed population of New Orleans, the proportion is small. Dr. Barton says:

"By the following table it will be seen, that notwithstanding the addition made to our mortality by emigrants and visiters with these diseases, yet we are more favored in these respects than any large city in this hemisphere.

| | Death from Phthisis to Total Mortality. | Death from all Pulmonary diseases to Total Mortality. |
|---|---|---|
| Philadelphia, | 14.84 pr. ct. | 28.57 pr. ct. |
| New York, | 17.50 " | 28.08 " |
| Havana, | 19.50 " | 25.07 " |
| Boston, | 15.13 " | 23.97 " |
| Baltimore, | 18.20 " | 23.33 " |
| Charleston, | 18.27 " | 22.73 " |
| Mexico, (city,) | 2.45 " | 16.76 " |
| Norfolk, | 11.01 " | 12.78 " |
| New Orleans, | 9.37 " | 13.87 " |

The aggregate mortality of New Orleans in 1849 was 9862, of which 29 per cent. were from deaths in the various hospitals.

"From this exhibit of the principal causes of mortality we proceed to refer you to the entire aggregate. It amounts to 9862; of whom, about 29 per cent. died in the various hospitals. From this, deducting 3176 for cholera, and 372 from causes of death other than *diseases*, the nett mortality amounts to 6314; being at a ratio of 1 in 16.67 or 5.99 per cent. The stationary population being estimated at 105,347; being an increase of 5.32 per cent., annually, over the population of 1847, when the census was taken."

The mortality in New Orleans in early life is very small, being only 36.98 per cent. in those under 20 years of age. That is to say, that for every 100 born, 63.02 reach their twentieth year. In Massachusetts the mortality is 40 per. cent., and in England 47 per cent., before the sixteenth year is reached.

The impression of the committee (Dr. Barton being the chairman and reporter,) is, that the mortality of New Orleans "is at *least double* what it ought to be, were such improvements made as science, observation and experience point out, and humanity and interest demand."

The second part of the report from which we have already made some excerpts, is devoted to a consideration of the means of improving the health of New Orleans, by a removal or mitigation of the causes of disease. These present themselves under the heads of "*great elevation of temperature*, [deficient] *ventilation*, *undue moisture and filth.*" Tables of mortality and of meteorology, and classification of the deaths and diseases of New Orleans, for the year 1849, accompany and give additional value to the report.

*Article Fifth*, by the Editor, is a "Special Report on the Fevers of New Orleans—particularly the Yellow Fever of 1849." He begins by telling of his opportunities for a practical acquaintance with the fevers of the country prior to his going to New Orleans to live, in 1841. Respecting the difference between these fevers and the yellow fever, and their proportionate prevalence in the city, Dr. Fenner thus discourses:

"In New Orleans we have met with all the forms of endemic fever which were familiar to us in the country, (West Tennessee, Mississippi and Madison Parish, La.,) with the addition of yellow fever and ship fever or genuine typhus. We have found those common to the city and county to prevail at the same season and in a similar manner, excepting that we meet with a more rapid and malignant congestive fever in the country than in the city, and the bilious remittents of the country retain their character throughout, more than they do in the city. Here, in the summer and autumn, they have a decided tendency to *crisis* by *hemorrhage.* This makes *yellow fever*—it forms the true characteristic difference between the high grades of summer and autumnal fever in the city and country, and *must depend on locality and dependent circumstances.* We have intermittent, remittent and continued fevers, alternating in type and running into each other, just as they do in the country. Intermittent fever prevails here throughout the year as it does in the country. During the healthiest years it predominates over all other types; but during the sicklier years, in the country, it runs into remittent bilious and congestive, whilst in the city it runs into yellow fever.

"Dr. Harrison testifies that he had often observed malignant intermittents immediately to precede the outbreaks of yellow fever epidemics.

"The New Orleans Charity Hospital is probably the most extensive fever hospital in the world. Let us see how far the statistics of disease at that institution will sustain our observations. It appears from the records, that in a period of *nine years*, from the 1st January, 1841, to 1st January, 1850, there were admitted into this hospital 73,216 patients; of which number there were admitted for *all the different forms of fever*, 33,381, (and among these last,) for *intermittent fevers*, 17,217.

"It would thus appear that nearly one-half of all the patients admitted into this hospital were for the different forms or types of *fevers*—and that *more than half of these were intermittents.*"

"The following statement will show the prevalence of intermittent fevers at the different seasons of the year, for the time specified:

| Int. Fever. | Spring. | Summer. | Autumn. | Winter. | All Fevers. | Yellow Fever. |
|---|---|---|---|---|---|---|
| 1841 | 112 | 403 | 177 | 92 | 1991 | severe. |
| 1842 | 114 | 453 | 394 | 135 | 1758 | little. |
| 1843 | 85 | 208 | 413 | 137 | 2222 | severe. |
| 1844 | 117 | 469 | 732 | 231 | 2207 | little. |
| 1845 | 180 | 353 | 664 | 206 | 1763 | none. |
| 1846 | 236 | 569 | 1045 | 218 | 2603 | little. |
| 1847 | 391 | 508 | 691 | 602 | 6901 | severe. |
| 1848 | 282 | 689 | 874 | 535 | 6361 | moderate. |
| 1849 | 420 | 1701 | 3738 | 1275 | 7575 | do. |
| Total | 2443 | 5353 | 7728 | 2331 | 3381 | |

"It will thus appear that intermittent fever prevails here all the year round; gradually increasing from the winter up to the autumn, when it begins to decline. It will be seen, however, that there is considerable variation in the amount, as well in the different seasons as in the different years. The proportion is greater in the healthy years; but intermittents are never entirely absent, even when yellow fever is raging. During the nine years specified, on selecting the month in which yellow fever was worst, we found the following relative proportion of intermittent fever at the Charity Hospital:

| | | | |
|---|---|---|---|
| 1841, September— | Yellow Fever | 682 | Intermittent 18 |
| 1842, " | " | 247 | " 144 |
| 1843, " | " | 365 | " 128 |
| 1844, " | " | 68 | " 255 |
| 1845, " | " | 00 | " 279 |
| 1846, October, | " | 83 | " 376 |
| 1847, August, | " | 1611 | " 74 |
| 1848, September, | " | 597 | " 299 |
| 1849, October, | " | 520 | " 720 |
| | | 4133 | 2293 |

Dr. Fenner says, "that, as all the forms of idiopathic fever met with at this locality *prevail together* and are frequently seen to *interchange, or run into each other*, we are led irresistibly to the conclusion that they are merely *modifications of diseases springing from one and essentially the same general remote cause.* All authors agree that the intermittent and remittent bilious fevers of the country, in the summer and early autumn, spring from the same remote cause, and consequently are but varieties and grades of the same disease. Now, we contend that the yellow fever of New Orleans holds the same relation to its intermittents, that the severe bilious fever of the country does to the intermittents there—they are therefore in the same category."

*Difference in their mode of termination.*—"The fevers of the country cause death by *inflammation of the brain* or of the *gastro-intestinal canal*, or by that strange lesion of the nervous system which is called *congestion*—the fevers of the city produce such an alteration of the blood and the solids as leads to fatal *hemorrhage* and *jaundice.*"

He warns us against continuing in the prevalent belief, that yellow fever is a disease of cities and towns, while intermittent and remittent fevers belong to the country. He has shown "that in New Orleans, one of the favorite abodes of yellow fever, we have all the forms of summer and autumnal fever met with anywhere—and yellow fever has been seen in very small villages, sometimes even in the country."

In other parts of the Report, more especially devoted to a consideration of yellow fever, Dr. Fenner states his having seen, during his attendance at the Charity Hospital in the months of September, October and November, cases of intermittent, mild remittent and dysentery run into well marked yellow fever. Without our engaging at this time in an examination of the *questio vexata*, the solution of which, to a certain extent, Dr. F. thinks he has reached, we would remark, that the appearance of diseases in a certain order of succession or concurrence, does not, by any means, establish their identity or even close affinity. The body may be rendered susceptible to an attack of yellow fever, as it may also of cholera, by morbid precursors and derangements of very various and even dissimilar kinds—a fit of indigestion, strong mental emotion, or even a fractured limb, for example.

Dr. Fenner claims somewhat of novelty for his views on the pathology and treatment of yellow fever. We could wish that they had been presented in a more definite and precise manner than we find them in the Report. Is the following meant to be a new view of the pathology of this disease? "In the early stage of yellow fever the derangement of the system is *entirely functional*, and consists chiefly in *lesion of innervation*. In the advanced stages it is altogether a different affair—*organic lesions have then taken place, and the blood is altered*."* The indications to be fulfilled in its treatment present nothing different from those of antecedent writers.

Touching the treatment, we want a larger addition of detailed facts before we can yield our convictions to the general statements of the editor. He had just been speaking of the circumstances under which sanguineous depletion is of use in yellow fever. "We merely wish to state that since 1847 we have learned from actual observation that liberal doses of quinine and opium, given *early in the disease, and during the exacerbation*, will subdue the fever and permanently and safely cut it short of its natural course, in a great many cases, without resorting to blood-letting in any manner." He quotes opinions, and iterates his own confidence in the free use of

* The italics in the extracts are all by the authors themselves.

quinine for the cure of yellow fever, "*mais mon cher ami*, you surely know that just the same eulogies have been lavished on the free use of the lancet, and on large doses of calomel, and on the *far niente* practice of some French and Spanish physicians, and yet, notwithstanding, people die in nearly the same proportion as before, of yellow fever." Were Dr. F. at our elbow, we would address him in this fashion—Will he not allow us the privilege of doing so at a distance?

Dr. J. C. Simonds furnishes Article VI., being "Statistics of Yellow Fever and of all Diseases in the Charity Hospital for thirty years, from 1820 to 1849 inclusive.

"The table shows for each year, and for each quinquenial period, the discharges, the deaths and their sum, and the mortality per cent. of yellow fever, the entire number of discharges and deaths of all diseases, and the proportion per cent. of yellow fever. The remarks are not based upon the figures in the table, which speak for themselves, but for the years 1820, 1822, 1824, have been obtained from a report of the Board of Health, and for the years from 1832 to 1844 from a paper by the late Dr. John Harrison, in which he states that 'the terms mild epidemic, epidemic, and violent epidemic, are intended to express degrees, both as to the prevalency and malignity of the disease.' 'In 1832 a violent epidemic of Asiatic Cholera raged at the same time the fever prevailed. In 1833, 1834 and 1835, there also existed sporadic cases of Cholera.'"

The seventh article is by the indefatigable editor, Dr. Fenner. It is a "Report on Epidemic Cholera in the City of New Orleans, 1848–'49." We read many interesting details in this paper, but none which would show that cholera derived any peculiar features from its abode in New Orleans, different from its well known character elsewhere. The questions of importation and home origin are taken up and illustrated by pertinent facts occurring in the field of Dr. Fenner's observations.

The almost entire exemption from the disease in the prisons and orphan asylums of the city, is worthy of note and remembrance.

"By reference to the reports of the orphan asylums and prisons of New Orleans, obligingly furnished by the attending physicians, it will be seen that cholera prevailed in only one asylum, (the Poydras Female,) and there only after the epidemic had completely subsided in the city, (see Dr. Rhodes' report.) The exemption of the inmates of prisons and asylums from epidemics of yellow fever, is a remarkable, but well established fact."

*Period of Greatest Severity.*—"The epidemic raged most severely from the 22d to the 30th of December, having reached its zenith about the 28th, on which day the deaths by cholera were *ninety-two.* From the 16th to the 22d, the weather was *oppressively warm*, the thermometer rising as high as 84°. From the 22d, it was cool, wet, gloomy, till the night of the 30th, when there fell a white frost. On the morning of the 1st of January, there was another white frost, and, from that time, the disease declined steadily."

*Comparison with the Epidemic of* 1832.—"The mortality from cholera, at its late visitation, compares most favorably with that of 1832, when it first scourged our city. The number of deaths by cholera, from the 12th of December, 1848, to the 20th January, 1849, as appears from the reports of the Board of Health, amounted to near fourteen hundred—five hundred and ninety-six of which occurred at the Charity Hospital. We learn from an interesting memoir on the cholera of 1832, addressed to the Academy of Medicine of Paris, by Dr. M. Halphen, a French practitioner of this city at that time, that the disease made its appearance about the 25th of October, in the midst of an epidemic of yellow fever; that, in a few days, it raged severely, and that, in the short space of twenty days, it killed about six thousand people. Dr. Halphen says, that the mortality amounted, on some days, as high as five hundred a day. He estimates the full population of the city then at fifty thousand; and, as cholera broke out during the prevalence of yellow fever, ere yet the absent citizens had returned, and before the customary visitors had dared to come in, he does not think the population, at that time, exceeded thirty-five thousand; thus showing the frightful loss of nearly *one-sixth of the population in about twenty days.* When we read over these sad details, we may well congratulate ourselves upon our happy deliverance from the late pestilence. True, we have lost about fourteen hundred people, and amongst them a few valuable citizens; but what would have been our fate, if so malignant a disease as that of 1832 had broken out in December last, when all of our own people were at home, and the city was full of strangers? In 1832, *the living could not afford decent burial to the dead.* Dr. Halphen states, that, on some days, upwards of one hundred corpses were accumulated at the cemeteries, awaiting interment. Large trenches were dug, into which cart loads of uncoffined bodies were heaped indiscriminately; and, in the dead of night, a great many bodies, with bricks and stones tied to their feet, were stealthily thrown into the river. The same ratio of mortality, at the present time, would demand about twenty thousand victims. Let us turn from the appalling calculation, and thank God that we have been so mercifully spared!"

We pass over the subject of Treatment, as presenting us with nothing new, although what is said is said plausibly.

On the subject of "premedication" as a part of prophylaxis we glean no encouragement from the trials, made at Dr. Fenner's suggestion.

Article eighth, also by Dr. Fenner, is a "Special Report on the Epidemic Colic which prevailed in the city of New Orleans during the summer of 1849."

More justice would have been done to the subject and to the author himself, if he had given precedence to a succinct account of the disease in New Orleans, instead of literary references and speculations as to its probable affinity with various forms of colic described by preceding writers. This blending of description of actual and recent phenomena with literary disquisitions only weakens the force of the former, and diminishes its historical value by distracting the attention of the reader.

"The cholic prevailed at New Orleans during the months of July, August and September; chiefly amongst the laboring class of whites; but to some extent amongst negroes. It occurred during a remarkably healthy season; though, in the time above mentioned, were to be seen cases of intermittent, remittent, typhoid and yellow fever, dysentery and diarrhœa. But these also were confined pretty much to the same classes, as shown by the records of the Charity Hospital. During the three months specified, the number admitted into this hospital was very great, whilst there was little doing in private practice."

No adequate cause of the colic is assigned. Hints are thrown out that it may be lead poison, arising out of the great extent of pipes made of this metal, by which the water is conveyed from the iron mains to the houses for domestic use. The length of leaden pipes now in use in New Orleans, is one million of feet, or more than 189 miles.

Free blood letting, anodynes and laxatives, aided by the warm bath and fomentations, constituted the best treatment in this epidemic colic.

Some useful remarks conclude this paper, on the tests for lead and copper in soda water; and also on lead poisoning, by Dr. Kittriedge of Cincinnati.

The ninth article of these Reports is "On the Topography, Climate and Diseases of the Parish of St. Mary, La., by James B. Duncan, M. D." This is a short but instructive paper, true to its title and promise.

The following is pertinent to an ethnographic question, now discussed with some animation by different writers:

"And here I will remark (as this subject is at the present time exciting much attention, that this class of people, including Terceroons, Quarteroons, Quinteroons, &c., in this vicinity, are as healthy, as robust, and as prolific as any portion of the community. Many of the men are fine specimens of manhood, have large families of children, and among them are now existing (both male and female) several remarkable instances of longevity. These remarks I presume may be confirmed by the testimony of other physicians practising among them."

Notice is taken of a common disease among negroes on plantations, the prominent symptom of which consists in *dirt-eating*. The condition of the system in which it manifests itself is, Dr. Duncan thinks, often produced by a deficiency of suitable nutriment.

"The diet of negroes on most plantations being salt pork, corn bread and molasses—rarely eating fresh meat and vegetables—a condition of the system is thus produced, closely allied to *scurvy*. In addition to the symptoms above described, I have occasionally seen a spongy state of the gums. There is generally present functional, and sometimes organic disease of the heart."

The treatment is well summed up in the following sentence: "Restore the healthy tone of the system, invigorate the subject, put rich blood into his veins, clothe him well, feed him well, and do not overtask him; arouse his feelings of pride, teach him to feel that he is a reasonable and rational being, and, in a majority of cases, success will attend our efforts, and we shall have the satisfaction of rescuing a valuable servant from the grave."

The next article is by Dr. Wm. A. Booth, "On the Cholera of Lafourche Interior." The editor expresses his opinion of its merits in quite emphatic language, when he asserts that it "contains the greatest number and variety of facts relative to cholera that has ever been published in America," so far as his knowledge extends. The various aspects of this awful scourge and its affinity to other diseases, are well stated. The same may be said of pathological phenomena exhibited in its course. But in all these we find no positive addition made to our prior knowledge of the subject; nothing assuredly depending on the manifestations of the epidemic in Lafourche.

"Reports on the Origin and Sanitary condition of the Orphan Asylums of New Orleans and Lafayette." By Drs. Rhodes, Carey and Sunderland.

A case of yellow fever has never been seen or heard of among the children in the "Poydras Female Orphan Asylum." The like exemption from cholera was not enjoyed. Out of 150 little inmates of the institution, "the whole number of cases of cholera could not have amounted to less than *fifty* or *sixty*." Why the precise number is not stated, we are at a loss to conjecture. Dr. Rhodes does not favor us with the number of deaths of those who were attacked. Their distribution in different directions, by the advice of Dr. R., need not have prevented him from procuring the requisite statistical returns.

Of two hundred children in the New Orleans Female Orphan Asylum, under the care of Dr. Carey, not a single case of cholera occurred in the establishment. There was one death, and two cases of children, with the disease on them when they were brought to the asylum.

Respecting the "Male Orphan Asylum of Lafayette," under the charge of Dr. Sunderland, we read:

"The average time that the boys have remained in the Asylum is two years, one month and twenty-three days. The average number of boys in the Asylum each year is 71.5. The average number of deaths each year 6.32, which is eight per cent. Of the children under seven years of age 14 per cent. die; while of those over seven only 3 per cent. have died.

"If we take into account only those children who were healthy when admitted, the mortality of those under seven years will be about five per cent., while that for those over seven will be about one per cent. per annum."

We are obliged from want of room, to postpone our notice of the remaining "Reports" until the August number. Their intrinsic worth makes us desirous of presenting a full analysis of them to our readers.

